# Recurrent implantation failure: a registry-corroborated, age-stratified definition and pragmatic five-tiered management algorithm

**DOI:** 10.3389/fendo.2026.1930159

**Published:** 2026-10-05

**Authors:** Hossam Elzeiny, Alexander M. Quaas, Alexandra P. Bielfeld, Michael F. Costello

**Affiliations:** 1 Reproductive Services Unit, City Fertility and The Royal Women’s Hospital, Melbourne, VIC, Australia; 2 Shady Grove Fertility Center, Solana Beach, CA, United States; 3 UniKiD – Department of Reproductive Endocrinology and Reproductive Medicine, University Medical Centre, Heinrich Heine University, Düsseldorf, Germany; 4 School of Clinical Medicine, University of New South Wales and Monash IVF Sydney, Sydney, NSW, Australia

**Keywords:** cumulative implantation rate, embryo transfer, endometrial receptivity, implantation, preimplantation genetic testing, recurrent implantation failure

## Abstract

Recurrent implantation failure (RIF) remains a significant challenge in reproductive medicine, imposing substantial emotional, clinical, and financial burden on affected couples. RIF is generally defined as failure to achieve pregnancy after multiple transfers of good-quality embryos, yet progress in the field has been constrained by the absence of a universally accepted definition and by the inherent complexity of investigating the embryo–endometrial interface. Recent evidence has prompted a reappraisal of the factors contributing to RIF, distinguishing it from the mechanistically distinct entity of embryo-intrinsic failure (recurrent embryo arrest and poor embryo development). This narrative review synthesises epidemiological data, evaluates the evidence base for available diagnostic modalities, and appraises current management strategies, including the controversies surrounding adjunctive treatments. We propose a registry-corroborated, age-stratified definition of RIF — the ABCDE framework — and a five-tiered, clinically applicable management algorithm that integrates the available evidence with expert recommendation to guide clinicians within a shared decision-making framework. Future directions include prospective multicentre validation, incorporation of artificial intelligence for risk prediction, and development of novel therapeutic approaches targeting the embryo–endometrial interface.

## Introduction

### The definitional problem

A live birth from *in vitro* fertilisation (IVF) is the result of an atraumatic transfer of a competent embryo capable of hatching and implanting into a receptive endometrium, followed by successful trophoblast invasion and placentation (1). When this process fails repeatedly despite the transfer of morphologically good-quality embryos, the clinical picture becomes one of the most difficult in reproductive medicine — and one of the least well-defined. Despite over two decades of research, the field has been unable to reach a consensus on how to define RIF (2). Definitions have evolved considerably over this period, from early criteria anchored to cleavage-stage embryo transfer number toward contemporary criteria incorporating blastocyst morphology and, more recently, euploidy status (3, 4); this progressive refinement reflects advancing embryological and genetic knowledge but has, paradoxically, further fragmented rather than converged consensus. More than 800 distinct definitions have been identified across approximately 5,700 published studies (5), rendering prevalence estimates unreliable, clinical comparisons invalid, and evidence synthesis incoherent.

This definitional disorder has direct clinical consequences. It drives premature investigation, sustains the widespread adoption of treatments without a validated evidence base, and — most consequentially — causes direct patient harm when embryo-intrinsic failure is systematically misclassified as a disorder of the implantation interface. The resulting misdirection of investigation towards endometrial and immunological factors, when the fundamental problem is embryonic, prolongs the diagnostic journey without therapeutic benefit.

### Clinical burden and prevalence

Infertility affects an estimated 17.5% of the adult population worldwide (6). More than 2.5 million IVF cycles are performed annually, generating approximately 500,000 live births (7, 8). Against this background, RIF represents a distinct and particularly challenging subpopulation carrying considerable emotional, financial, and clinical burden for affected patients and their families.

### Aims and scope of this review

This narrative review provides a structured critical appraisal of twelve commonly requested investigations in recurrent implantation failure and illustrates the ABCDE framework through three de-identified clinical cases. The twelve investigations selected for appraisal represent those most frequently requested by referring clinicians and most commonly encountered in tertiary RIF consultation, informed by international survey data on clinician and embryologist practice patterns (142) together with the authors’ collective tertiary referral experience.

### Literature search strategy

This narrative review was based on a literature search conducted in PubMed/MEDLINE and EMBASE, covering studies published between January 2000 and May 2026. Search terms included “implantation failure”, “recurrent implantation failure”, and “RIF”, combined with “immunology”, “implantation window”, “endometrial receptivity”, “preimplantation genetic testing”, “adenomyosis”, “chronic endometritis”, and “cumulative live-birth rate”, together with the corresponding Medical Subject Headings (MeSH). Inclusion criteria comprised full-length, peer-reviewed, English-language articles reporting original data, systematic reviews, meta-analyses, or consensus statements relevant to RIF pathophysiology, diagnosis, or management, with studies involving euploid embryo transfer prioritised where available. Exclusion criteria comprised conference abstracts, non-peer-reviewed preprints, case reports of fewer than five patients, and non-English-language publications without an available translated full text. Systematic reviews, meta-analyses, and randomised controlled trials were prioritised, supplemented by expert consensus statements, registry analyses, and landmark pre-2020 publications where essential for historical or conceptual context. ABCDE framework thresholds were derived from aggregate data published by SART, ANZARD, and ESHRE. The review draws on 162 peer-reviewed references.

## Epidemiology and the prevalence problem

Infertility affects an estimated 17.5% of the adult population worldwide (6, 7). The most recent ICMART report, encompassing data from 2,746 fertility centres across 76 countries, recorded approximately 1.93 million ART cycles — representing 66% of estimated global activity — resulting in 439,039 deliveries (8). Definitions of RIF have evolved with advancements in ART. Earlier criteria based on cleavage-stage embryo transfers (3) have been replaced by more rigorous criteria focusing on euploid blastocyst transfers (4). The adoption of PGT-A-enabled deselection of aneuploid embryos, yet its availability remains limited by financial, regulatory, and cultural barriers in some regions (9).

The lack of consensus has led to variability in prevalence estimates (2, 5). Reported incidences of biochemical pregnancy loss (BPL) range from 14% to 18% in ART populations (10, 11). Busnelli et al. (12) estimated that approximately 10–15% of ART patients experience RIF, whilst Kolanska et al. (13) reported that RIF affects nearly 50% of couples undergoing ART. Population-based study of linked IVF cycle data reported that cumulative pregnancy rates exceeding 80% after six treatment cycles suggest that fewer than 20% of patients experience true RIF (14, 15). Pirtea et al. (16) reported a <5% prevalence after three consecutive euploid embryo transfers, and Gill et al. (17) observed a prevalence below 2% after five consecutive euploid transfers in 123,987 patients, challenging earlier assumptions and even the existence of RIF as a distinct phenomenon (18). This wide variation in reported prevalence (4–50%) chiefly reflects heterogeneous underlying definitions — cleavage-stage versus blastocyst-stage transfers, tested versus untested embryos, biochemical versus clinical pregnancy endpoints, and differing numbers of qualifying transfers required — rather than genuine population differences.

## Pathophysiology

RIF arises from synergistic contributions of embryonic, anatomical, immunological, lifestyle, and environmental factors (**Figure 1**). For clarity and brevity, these are consolidated below into four thematic groups.

**Figure 1:**
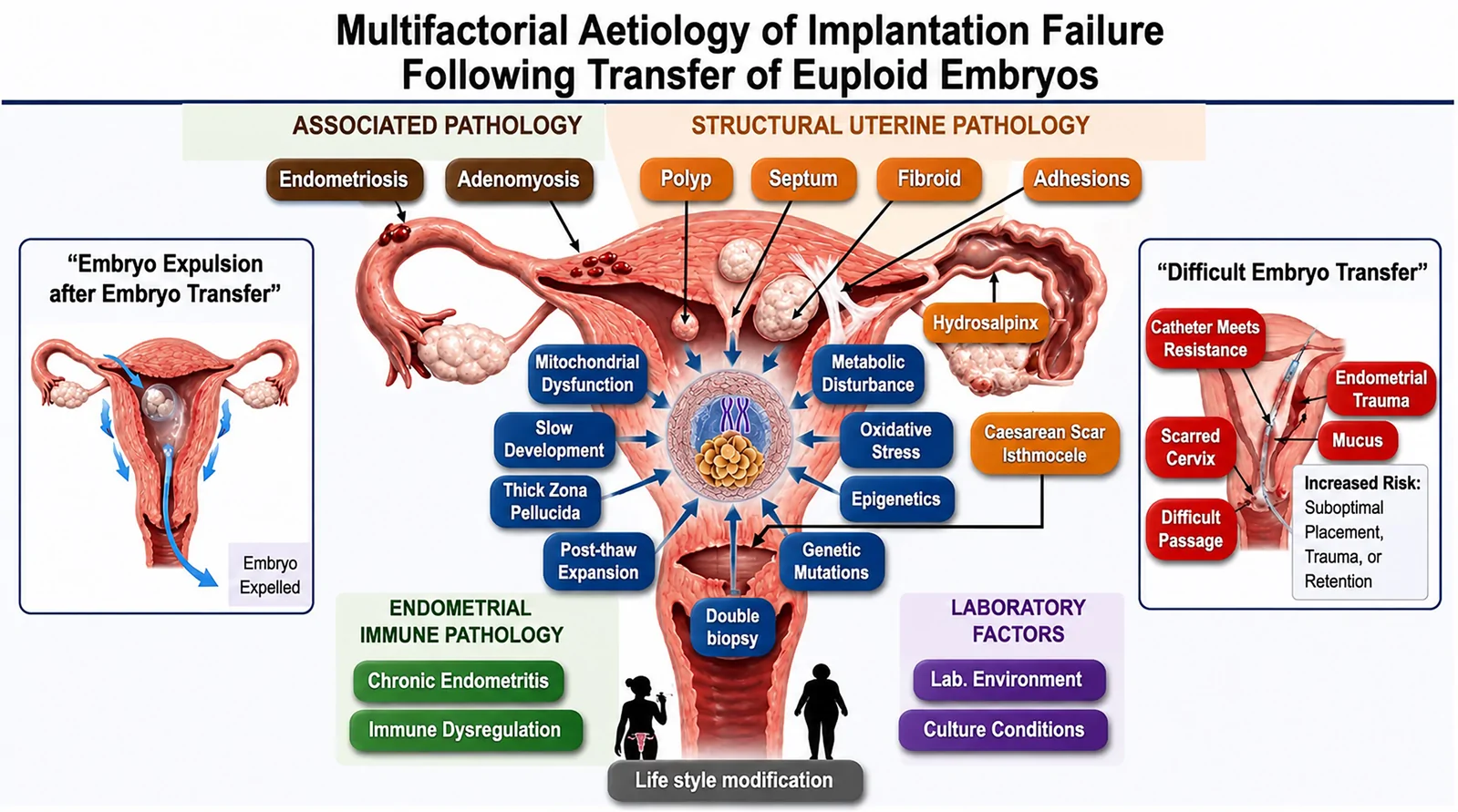
Multifactorial determinants of recurrent implantation failure (RIF) in euploid embryo transfer. Embryonic contributors include genetic and epigenetic alterations, oxidative stress, mitochondrial dysfunction, metabolic disturbances, double trophectoderm biopsy, thick zona pellucida, timing of blastocyst development, post-thaw re-expansion, and suboptimal laboratory or culture conditions. Anatomical causes encompass hydrosalpinx, endometrial polyps, intrauterine adhesions, uterine septa, caesarean isthmocele, fibroids, embryo expulsion, technique of embryo transfer, endometriosis, and adenomyosis, each capable of impairing endometrial receptivity or embryo apposition. Immunological factors, together with alterations in the endometrial microbiome and chronic endometritis, further contribute through immune dysregulation. Collectively, these domains illustrate the complex, interdependent pathways governing implantation success. RIF, recurrent implantation failure.

### Embryonic and genetic factors

Embryonic chromosomal imbalance remains the dominant embryo-intrinsic cause of implantation failure: most autosomal monosomies, haploidy, and polyploidy are negatively selected before blastocyst formation, while mosaic embryos show implantation rates that fall with increasing level of mosaicism (19–21).

Beyond conventional aneuploidy, single-gene disorders, mitochondrial mutations, and loss-of-function variants affecting angiogenesis, immune modulation, hormonal signalling, cell adhesion, and cytoskeletal organisation constitute an increasingly recognised molecular substrate for implantation failure in euploid embryos (22, 23).

Epigenetic modifications — DNA methylation, histone remodelling, and non-coding RNA expression — are indispensable for normal embryogenesis and endometrial receptivity; infertility per se is independently associated with widespread embryonic epigenetic dysregulation, distinct from any ART-related perturbation (24, 25).

Oxidative stress, reflecting an imbalance between reactive oxygen species and antioxidant defence, impairs endometrial receptivity and promotes embryonic DNA damage, though excessive antioxidant activity is equally detrimental, underscoring the importance of redox homeostasis over simple supplementation (26, 27).

Trophectoderm mitochondrial DNA content has been proposed as a marker of bioenergetic status, though evidence for its correlation with live-birth rates remains inconsistent (28–30). Metabolically quiet embryos investing energy solely in growth are associated with higher implantation competence than metabolically noisy embryos generating signals that destabilise decidualised stromal gene expression (31, 32).

Double trophectoderm biopsy and double vitrification are each associated with reduced sustained implantation and higher miscarriage risk in systematic review, although double vitrification alone does not appear to compromise outcomes in unbiopsied embryos (33, 34). Zona pellucida hardening — exacerbated by *in vitro* culture — may mechanically impede blastocyst hatching despite morphological competence (35).

Delayed blastocyst formation (day 6 or 7) is associated with lower euploidy rates and inferior implantation potential even after artificial-intelligence-assisted selection (36, 37), and post-thaw blastocoel re-expansion within the first two hours after warming is itself a biomarker of viability, with poor re-expansion and multiple blastocyst collapse independently associated with reduced implantation and increased aneuploidy risk (38, 39).

### Structural and anatomical factors

Structural uterine and tubal pathology remains among the more tractable contributors to implantation failure.

Endometrial polyps are present in approximately one-third of women undergoing hysteroscopic evaluation before IVF, with pregnancy rates after polypectomy varying by polyp size and number (40, 41); Removal is recommended in infertile women generally and is specifically justified in RIF (42).

Hydrosalpinx is strongly associated with RIF through NF-κB-mediated downregulation of CFTR and MUC1, impaired pinopode formation, and direct embryotoxic effects of tubal fluid; salpingectomy or proximal occlusion reverses these effects and restores IVF outcomes (43, 44).

Uterine septum, the most prevalent congenital uterine malformation, is linked to miscarriage, preterm delivery, and malposition, with surgical resection substantially improving pregnancy outcomes; the T-shaped uterus represents an additional morphological variant associated with impaired implantation (45, 46).

Caesarean scar niche (isthmocele) correlates with reduced implantation, pregnancy, and live-birth rates, plausibly via fluid accumulation and chronic endometritis, although the evidence is divided regarding euploid transfer outcomes specifically in the absence of intracavitary fluid (47, 48).

Fibroids impair endometrial function through direct cavity distortion, aberrant angiogenesis, and disruption of the endometrial microbial environment; non-cavity-distorting intramural fibroids of 2–6 cm have a demonstrable deleterious effect on live-birth rates (49, 50).

### Endometrial and immunological factors

Endometriosis and adenomyosis converge on a shared molecular mechanism: increased local intra-endometrial oestrogen driving progesterone-receptor resistance, with aromatase upregulation, pro-inflammatory cytokine expression, and impaired HOXA10 expression in the eutopic endometrium (51). The clinical signal attributable to endometriosis per se is confounded substantially by reduced ovarian reserve and co-occurring adenomyosis; well-controlled matched studies demonstrate equivalent live-birth rates between women with and without endometriosis once these factors are accounted for, and endometriosis does not appear to impair live-birth rates in frozen transfers of euploid blastocysts specifically (52).

Adenomyosis, by contrast, is more consistently associated with reduced live-birth and increased miscarriage rates, including in euploid transfer cycles, with the effect most pronounced when lesions involve the inner myometrium (53–55).

Implantation is further governed by a precisely choreographed immunological shift at the maternal–fetal interface: extravillous trophoblasts expressing HLA-C, HLA-G, and HLA-E interact with uterine NK cells, which constitute approximately 70% of decidual leucocytes in the mid-luteal phase, while a transition from Th1 to Th2 cytokine dominance and macrophage polarisation from M1 to M2 supports tolerance and spiral-artery remodelling; dysregulation of this balance has been linked to recurrent pregnancy loss and RIF (56–61).

### Laboratory and modifiable systemic factors

Laboratory and culture conditions constitute a final, modifiable determinant. Suboptimal culture conditions, volatile organic compounds, and media-specific transcriptomic and metabolic signatures each disrupt epigenetic programming and implantation potential, while autologous endometrial co-culture has been shown to improve blastocyst development in a randomised trial (62–66).

Modifiable patient-level risk factors — smoking, alcohol, obesity, and psychological stress — act through overlapping endocrine, metabolic, and inflammatory pathways and their optimisation is a tier-one clinical priority; smoking is associated with significantly reduced live birth per IVF cycle (OR 0.59, 95% CI 0.44–0.79) and increased miscarriage risk, while maternal anxiety impairs embryo implantation via downregulation of decidual adrenergic receptor signalling (OR 2.22, 95% CI 1.10–4.48) (67–70).

## Diagnostic precision: defining RIF and distinguishing it from its mimics

It is paramount to distinguish between recurrent implantation failure (RIF) and recurrent IVF failure (71), as recurrent IVF failure encompasses two distinct subgroups: RIF — a disorder of the implantation interface — and embryo-intrinsic failure (recurrent embryo arrest and poor embryo development), a disorder of embryo quality. This distinction, and its application within the ABCDE framework, is illustrated in **Table 1**.

**Table 1:** Distinguishing recurrent implantation failure (RIF) from recurrent IVF failure: illustrative clinical cases.

Case	Clinical course	ABCDE classification and rationale
Ms S.C. 31 years	Presented with recurrent IVF failure in the context of her partner’s azoospermia secondary to cystic fibrosis carrier status. Four IVF cycles at another centre: cycles 1–2 yielded 8 and 10 vitrified oocytes; cycle 3, 18 oocytes thawed (5 survived), ICSI with fresh microTESE sperm yielded a single fertilised oocyte and a day-3 embryo transfer, no pregnancy; a further cycle using frozen sperm had total fertilisation failure; a subsequent cycle, physiological ICSI with testicular aspiration sperm, 3/7 oocytes fertilised, an early blastocyst transferred, low βhCG not progressing. Care was transferred and a fifth cycle undertaken: 21 oocytes, 14 fertilised (fresh modified unilateral open TESE sperm with artificial oocyte activation), 10 high-quality blastocysts generated (6 day-5, 4 day-6). A single 5AA blastocyst was transferred; serum βhCG 512 IU/L confirmed pregnancy, culminating in spontaneous vaginal delivery of a healthy girl at 39 weeks (2.6 kg).	*Recurrent IVF failure, not RIF: only two embryo transfers occurred across the four outside cycles (one day-3 cleavage-stage transfer, one early blastocyst transfer), neither meeting ABCDE’s good-quality blastocyst threshold. Illustrates the distinction between recurrent IVF failure and true RIF.*
Ms P.P. 38 years	Primary infertility of 26 months. First stimulation: 8 oocytes, 7 fertilised, 4 day-6 blastocysts; two untested FETs unsuccessful. The remaining two embryos underwent PGT-A: one euploid, one trisomy 22. Transfer of the euploid embryo failed. A second IVF cycle yielded 12 oocytes, 8 fertilised, 3 blastocysts (2 day-6, 1 day-7), all euploid. A further day-6 euploid FET (GnRH-agonist artificial endometrial preparation) failed. Care was transferred; hysteroscopy with dilation and curettage was performed. A subsequent FET (GnRH-agonist hormone-replacement protocol with intensive luteal-phase progesterone) transferring two embryos (day-6 and day-7) resulted in pregnancy (βhCG 273 IU/L), a viable singleton on ultrasound, and caesarean delivery of a healthy boy at 39 weeks (2.645 kg).	*Meets ABCDE RIF criteria via the euploid pathway: two failed euploid transfers, satisfying the compressed threshold of two failed euploid transfers irrespective of age.*
Ms R.O. 39 years	Longstanding severe stage IV endometriosis; laparoscopic excision of rectal nodules and ovarian endometriomas followed by three months of GnRH-agonist therapy. A first cycle was cancelled for ovarian over-response. A second cycle generated 4 blastocysts, none of which implanted. A third cycle included four further FETs without pregnancy — 8 failed good-quality blastocyst transfers in total. Care was transferred. A fourth cycle yielded 14 oocytes; 9 blastocysts underwent PGT-A (1 aneuploid, 2 day-6 mosaic, 6 euploid: 3 day-5, 3 day-6). Subsequent double embryo transfers produced two biochemical pregnancies, one anembryonic gestation, and no pregnancy from the final two mosaic embryos. Progressively enlarging intramural fibroids (not distorting the cavity) prompted abdominal myomectomy and adenomyomectomy. A fifth cycle yielded 11 oocytes, with 4 blastocysts cryopreserved without PGT-A. Transfer of a single 4AA day-5 embryo (GnRH-agonist and letrozole protocol) with adjunctive therapy resulted in pregnancy (positive serum βhCG) and caesarean delivery of a healthy boy at 38 + 4 weeks (2.860 kg).	*Definitive RIF: 16 unsuccessful embryos transferred in total, including six euploid embryos, across eight years of infertility — illustrating the framework’s application at its most clinically demanding end.*

ABCDE, Age/Blastocyst quality/Cycle number/Degree of expansion/Euploidy; FET, frozen embryo transfer; ICSI, intracytoplasmic sperm injection; PGT-A, preimplantation genetic testing for aneuploidy; RIF, recurrent implantation failure; TESE, testicular sperm extraction.

Conflating these entities fundamentally misdirects clinical investigation and exposes patients to inappropriate, costly, and potentially harmful interventions; the two terms are not synonymous and should never be used interchangeably (71). Throughout this manuscript we refer to the latter subgroup descriptively as embryo-intrinsic failure, rather than as a proposed formal diagnostic category. Directing investigation towards endometrial receptivity or immunological dysregulation in patients with embryo-intrinsic failure is clinically inappropriate when the fundamental problem is embryonic — as illustrated concretely by Case Ms S.C. above, whose four cycles at another centre yielded only two qualifying transfers, neither meeting the ABCDE blastocyst-quality threshold; had this patient instead been referred for endometrial receptivity or immunological testing, recognition of the true underlying problem — severe male-factor-related fertilisation and embryo development, ultimately resolved by a modified fertilisation approach — would have been delayed without therapeutic benefit.

The ESHRE Working Group on RIF defines the condition as repeated failure to achieve a positive pregnancy test following transfer of embryos considered viable (72). This definition correctly localises RIF at the implantation interface but requires operationalisation to be clinically useful; the ABCDE (Age/Blastocyst quality/Cycle number/Degree of expansion/Euploidy) framework proposed here provides that operationalisation. The serum beta-human chorionic gonadotrophin (βhCG) test, rather than the ultrasound-detected gestational sac, is the correct diagnostic endpoint, as biochemical pregnancies represent implantation without sustained development (71). Because the ABCDE diagnostic endpoint is sustained implantation, biochemical pregnancies — representing implantation without development — count as failed transfers for threshold-counting purposes. Misclassifying their absence as successful implantation artificially inflates the transfer threshold required to reach an RIF diagnosis.

### The cumulative implantation rate threshold and the 80% benchmark

Establishing a cumulative implantation rate (CIR) threshold is essential for anchoring the RIF definition to population-level data and avoiding both premature and delayed investigation. Two thresholds have been proposed: the ESHRE Working Group suggested 60% (72), whilst the Lugano Workshop recommended a considerably stricter threshold of 95% (18). Neither is without limitation. A pragmatic and biologically grounded alternative is to align the CIR threshold with the cumulative natural conception rate after 12 months of unprotected intercourse in the general population — approximately 80–85% (73). We emphasise at the outset that this 80% anchor is a considered consensus benchmark, supported by biological analogy with natural fertility, rather than a mathematically derived optimum; a formally data-derived optimal threshold remains an open question and is listed among the research priorities in the Discussion. An 80% threshold is clinically defensible on three grounds: it exceeds the ESHRE threshold sufficiently to exclude statistical variation as the predominant explanation for repeated failure; it avoids the impracticality of the 95% threshold, which would require excessive transfer numbers incompatible with patient retention; and it aligns with the natural fertility benchmark that justifies initiating investigation in spontaneously conceived pregnancies.

## The ABCDE framework: derivation, parameters, and cross-registry corroboration

### Framework parameters

The ABCDE framework integrates five parameters, each addressing a distinct determinant of implantation potential (**Figure 2**). All five must be satisfied for a transfer to count toward the diagnostic threshold. We use the term “corroboration” throughout this manuscript specifically to denote the convergence of independently derived aggregate calculations across three registries; this is categorically distinct from, and does not substitute for, prospective validation in an independent individual-level cohort — a distinction we make explicit here, at first use, and reiterate in the Discussion.

**Figure 2:**
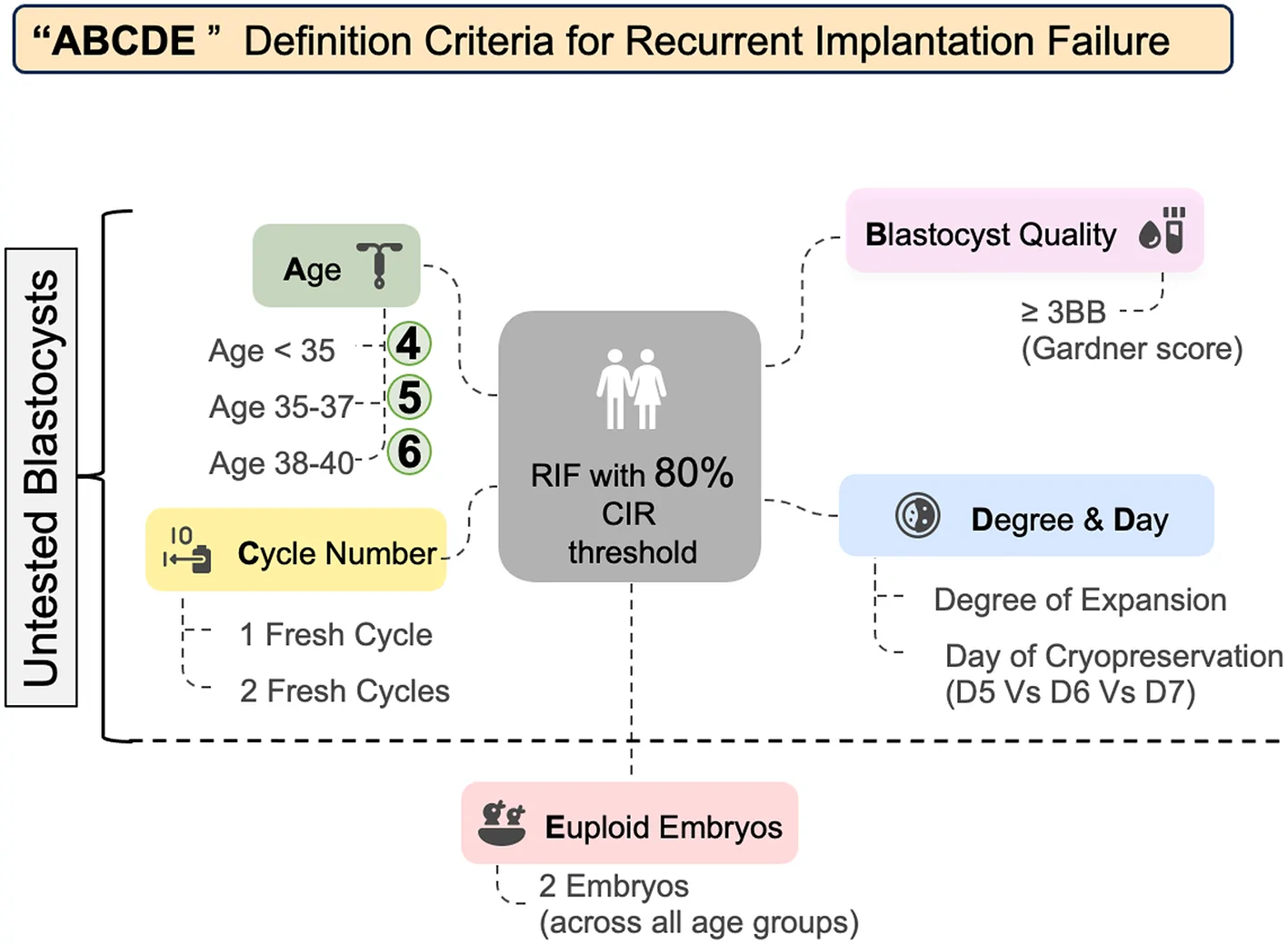
The ABCDE framework: an evidence-based diagnostic model for recurrent implantation failure (RIF) calibrated to an 80% cumulative implantation rate threshold. The framework incorporates Age, Blastocyst quality, Cycle number, Degree of expansion and vitrification day, and Euploidy status. For untested embryos, the first four parameters apply; age strata (<35, 35 to 37, and 38 to 40 years) and the corresponding number of failed embryo transfers define RIF thresholds. Failure of two euploid embryo transfers meets RIF criteria irrespective of age. Individual-level 95% confidence intervals are not available at the aggregate registry level from which these thresholds were derived. ABCDE, Age/Blastocyst quality/Cycle number/Degree of expansion/Euploidy; CIR, cumulative implantation rate; RIF, recurrent implantation failure.

- Age is the dominant determinant of implantation potential. Stratification into three groups — younger than 35, 35–37, and 38–40 years — defines age-specific thresholds. Women older than 40 years are managed with individualised counselling regarding oocyte quality rather than a standard RIF threshold (74).
- Blastocyst quality specifies that qualifying transfers must involve good-quality blastocysts (75). Transfer of poor-quality blastocysts does not count toward the ABCDE threshold. A patient whose entire transfer history consists of cleavage-stage embryos, for example, does not meet criterion B at any point and therefore does not qualify for an ABCDE RIF diagnosis regardless of transfer number, falling instead into the recurrent IVF failure category illustrated by Case Ms S.C. (**Table 1**).
- Cycle number recognises that qualifying embryos may derive from a single stimulation cycle in patients with a robust ovarian response (yielding 3–5 qualifying blastocysts) or from up to two cycles in normal responders (76, 77). The failed-transfer count is tallied at the level of individual embryo transfers, irrespective of how many transfers derive from a single stimulation cycle, provided the qualifying embryos originate from no more than the permitted number of cycles for that patient’s ovarian-response category. For example, three sequential single-embryo transfers arising from one high-responder stimulation cycle count as three failed transfers toward the relevant age-stratified threshold.
- Degree of expansion and Day of vitrification acknowledges that blastocysts vitrified on day 5 with adequate post-thaw re-expansion yield substantially superior outcomes compared with day-6 embryos or those with poor post-thaw recovery (37, 38). Day-7 blastocysts are not excluded from the ABCDE framework but are tracked and reported separately, given their independently lower euploidy rates and the evidence that post-thaw outcomes are vitrification-day dependent (78, 79); to avoid ambiguity, we clarify that a failed day-7 transfer of an otherwise qualifying blastocyst still counts toward the relevant threshold — it is flagged separately in reporting, not excluded from threshold-counting.
- Euploidy: Where preimplantation genetic testing for aneuploidies (PGT-A) has been performed, the diagnostic threshold is markedly compressed to two failed euploid transfers, as embryo aneuploidy — the principal source of age-associated implantation failure — has been eliminated prior to transfer (16, 17). The derivation of this threshold is described below.

### Statistical derivation of age-stratified thresholds for untested embryos

Under the assumption of independent and identically distributed (i.i.d.) implantation probabilities across successive transfers (4, 80), the minimum number of failed transfers n required to exceed an 80% cumulative probability of implantation is given by n = ln (1 − 0.80) ÷ ln (1 − p), where p is the per-transfer biochemical pregnancy rate for a given age stratum, rounded up to the nearest integer. This i.i.d. assumption represents a recognised modelling approximation: per-transfer implantation rates demonstrably decline across successive cycles, such that the derived thresholds represent pragmatic clinical heuristics anchored to population-level data rather than precise individual probability estimates. Biochemical pregnancy rates were extracted from SART (74), ANZARD (81), and ESHRE (**Table 2** footnote); where registries report clinical pregnancy or live-birth rates as primary outcomes, these were used as conservative proxies, such that the derived thresholds may be marginally conservative relative to a model using true biochemical pregnancy rates. Results are presented in **Table 2**.

**Table 2:** Cross-registry derivation of ABCDE age-stratified transfer thresholds.

Registry	Age (years)	p (per-transfer implantation rate)	Rounded n	ABCDE threshold
SART (USA)	<35	0.40	4	4
SART (USA)	35–37	0.34	4	5†
SART (USA)	38–40	0.24	6	6*
ANZARD (Aus/NZ)	<35	0.41	4	4
ANZARD (Aus/NZ)	35–37	0.36	4	5†
ANZARD (Aus/NZ)	38–40	0.26	6	6*
ESHRE (Europe)	<35	0.40	4	4
ESHRE (Europe)	35–37	0.34	4	5†
ESHRE (Europe)	38–40	0.24	6	6*

†Adopted on clinical grounds. *Adopted as mathematically derived and cross-registry corroborated. n = ln(1 – 0.80) ÷ ln(1 − p), rounded up to the nearest integer. ABCDE, Age/Blastocyst quality/Cycle number/Degree of expansion/Euploidy; ANZARD, Australia and New Zealand Assisted Reproduction Database; CIR, cumulative implantation rate; ESHRE, European Society of Human Reproduction and Embryology; SART, Society for Assisted Reproductive Technology. ESHRE per-transfer rates derived from European IVF Monitoring (EIM) Consortium data (European IVF Monitoring Consortium (EIM) for ESHRE; Wyns C, De Geyter C, Calhaz-Jorge C, et al. ART in Europe, 2018: results generated from European registries by ESHRE. Hum Reprod Open. 2022;2022(3):hoac022), aligned to SART age strata for comparison.

### Statistical derivation of euploid embryo thresholds

Pirtea et al. (16) reported per-transfer sustained implantation rates of 69.9%, 59.8%, and 60.3% across three consecutive frozen euploid single embryo transfers in 4,429 women, yielding a cumulative sustained implantation rate of 95.2% and a cumulative live-birth rate of 92.6% after three transfers; biochemical pregnancies were explicitly excluded from these outcome metrics. Gill et al. (17), in a multicentre retrospective study of 123,987 patients, demonstrated that only 105 patients (0.085% of the screened population) proceeded to a further transfer after three consecutive failed euploid transfers, providing epidemiological corroboration that true unexplained implantation failure after euploid transfer is rare, with a population prevalence below 2%. Applying the binomial model n = ln(1 − 0.80) ÷ ln(1 − p) with the conservative lower-cycle per-transfer rate of p = 0.60 yields n = 1.76, rounded up to 2; the first-transfer rate of p = 0.699 yields n = 1.34, also rounded up to 2. The threshold of two failed euploid transfers is therefore arithmetically robust across the per-transfer rates directly reported in the source dataset, and is adopted as the ABCDE “E” criterion for women up to and including 40 years of age, once embryo aneuploidy — the principal source of age-associated statistical variation — has been eliminated by PGT-A.

We note the apparent tension between this threshold of two and the markedly lower observed prevalence of true failure after three or more euploid transfers reported by Gill et al. (0.085%): the threshold of two is deliberately derived from the per-transfer implantation-rate binomial model rather than from Gill et al.’s descriptive population prevalence, and represents an intentionally earlier trigger point that prioritises timely investigation over a small increased risk of premature workup — a considered trade-off rather than an assumption-free optimum. Prospective comparison of two- versus three-transfer euploid thresholds is listed among the research priorities in the Discussion.

### Threshold stability assessment

Threshold stability was assessed by perturbing p by ±5 percentage points for each age stratum; this is an arithmetic perturbation exercise applied to the aggregate model inputs, rather than a formal statistical sensitivity analysis incorporating uncertainty in the underlying registry data, and formal uncertainty quantification awaits prospective individual-level cohort data.

Perturbation results were stable for the <35 and 35–37 strata, with adopted thresholds remaining at or within one transfer of the mathematical ceiling. The 38–40 age stratum was most sensitive to perturbation, reflecting the steeper age-related decline in per-transfer implantation rates; this sensitivity profile is acknowledged as a limitation and underscores the priority of prospective individual-level validation in this age group.

### Cross-registry convergence

Threshold calculations converge across three independent international registry datasets. SART and ESHRE data yield mathematically identical rounded n values of 4, 4, and 6 across women aged <35, 35–37, and 38–40 years respectively; ANZARD yields closely concordant values of 4, 4, and 6 across comparable age strata (**Table 2**). The adopted ABCDE thresholds of 4, 5, and 6 failed transfers for these three age groups represent a clinically intuitive age-graduated progression anchored to population-level registry data from three independent international sources.

It is explicitly acknowledged that cross-registry corroboration of aggregate binomial calculations does not constitute prospective external validation in an independent individual-level cohort; that step is the stated prerequisite for guideline adoption and the primary target for future research.

## Investigations in recurrent implantation failure

A pre-IVF assessment encompassing ovarian reserve testing, transvaginal ultrasonography, and semen analysis represents the foundation of the infertility workup. In the context of RIF, this baseline assessment must be repeated and reviewed critically before any additional investigations are initiated. **Table 3** presents a structured critical appraisal of 12 commonly requested investigations, formally separating evidence-supported from non-evidence-based modalities; a Supplementary Table provides the specific reference(s) and justification underpinning each assessment shown.

**Table 3:** Critical appraisal of twelve commonly requested investigations in recurrent implantation failure.

Investigation	Biological plausibility	Association	Causation	Threshold	Evidence	Reproducibility	Accuracy	Standardisation	References
Uterine/vaginal microbiome	✗	✗	✗	✗	✗	✗	✗	✗	(103, 104)
NK cells	✓	✓	✗	✗	✗	✗	✗	✗	(105, 106)
ERA test	✓	✗	✗	✗	✗	✗	✗	✗	(107, 108)
Thrombophilia	✓	✗	✗	✗	✗	✓	✓	✓	(109, 110)
HLA-DQ	✓	✓	✗	✗	✗	✗	✗	✗	(111)
Immune testing	✓	✓	✗	✗	✗	✗	✗	✗	(72)
Coeliac screening	✗	✗	✗	✗	✓	✓	✓	✓	(116–120)
ANA & ATA	✓	✗	✗	✗	✗	✓	✗	✓	(116–122)
Sperm DNA fragmentation	✓	✓	✗	✗	✗	✗	✗	✗	(112–114)
Endometrial function test	✓	✗	✗	✗	✗	✗	✗	✗	(115)
KIR-HLA genotype	✓	✓	✗	✗	✗	✗	✗	✗	(123, 124)
Vitamin D level	✓	✓	✗	✗	✗	✓	✗	✓	(125–127)

✓, criterion met; ✗, criterion not met. Criteria are defined operationally as follows: Biological plausibility — a coherent mechanistic pathway has been proposed in the literature; Association — at least one study reports a statistically significant relationship with implantation or pregnancy outcome; Causation — prospective or interventional evidence supports a causal rather than correlative relationship; Threshold — a clinically validated diagnostic cut-off exists; Evidence — supportive data derive from RCT or meta-analysis rather than observational study alone; Reproducibility — findings have been independently replicated; Accuracy — reported sensitivity/specificity or diagnostic performance meets an accepted standard; Standardisation — a validated, consensus laboratory or reporting protocol exists. None of the twelve investigations currently has sufficient evidence to support routine clinical use; these conclusions reflect the present evidence base and expert consensus, although selected tests may warrant reconsideration as methodological rigour and standardisation improve. ANA, antinuclear antibodies; ATA, antithyroid antibodies; ERA, endometrial receptivity assay; HLA-DQ, human leucocyte antigen-DQ; KIR-HLA, killer immunoglobulin-like receptor-HLA; NK, natural killer cells.

### Evidence-supported investigations

Three-dimensional pelvic ultrasonography offers superior diagnostic accuracy for uterine cavity abnormalities (97.6% versus 86.6% for two-dimensional assessment) and may be considered in RIF (82, 83).

Hysteroscopy remains the gold standard for uterine cavity evaluation, enabling detection of subtle abnormalities missed by ultrasound and targeted biopsy for chronic endometritis, with meta-analyses suggesting modest improvement in pregnancy outcomes (84–86).

Laparoscopy reveals previously undetected pelvic pathology — endometriosis, adhesions, tubal abnormality — in a substantial proportion of patients, and combined hysteroscopy–laparoscopy provides the most comprehensive single-session assessment (87–90).

Parental karyotyping should be considered in couples with unexplained implantation failure, particularly given the two- to fourfold increase in translocation prevalence relative to the general population (2.5% versus 0.6%); targeted testing following recurrent euploid-transfer failure, rather than universal testing at first RIF presentation, may represent a more cost-effective triage strategy warranting dedicated economic analysis (91–93).

Preimplantation genetic testing for aneuploidy (PGT-A) is the pivotal tier-two investigation: women with RIF demonstrate 43–79% higher embryo aneuploidy rates than fertile controls, and PGT-A serves a diagnostic rather than interventional role by deselecting aneuploid embryos and directing resources toward euploid embryos of higher implantation potential and shorter time to pregnancy without itself increasing cumulative live birth (94–100).

### Non-evidence-based investigations: a critical appraisal

A substantial number of investigations are routinely offered to patients with RIF despite a lack of evidence supporting clinical utility (**Table 3**), increasing costs, risking overdiagnosis, and occasionally leading to inappropriate treatment (101, 102). These include uterine and vaginal microbiome testing, limited by lack of standardisation and sampling contamination (103, 104); peripheral (as opposed to uterine) natural killer cell testing, which does not reliably reflect endometrial NK activity (105, 106); the endometrial receptivity assay, for which the largest RCT to date found no live-birth difference compared with standard timing, alongside evidence of substantial intra-patient cycle-to-cycle variability (107, 108); thrombophilia screening, HLA-DQ alpha genotyping, sperm DNA fragmentation testing, and endometrial immune profiling panels, none of which are currently supported by randomised evidence (109–115); coeliac disease and thyroid autoimmunity screening, neither of which affects live-birth rates in the absence of overt dysfunction (116–120); antinuclear antibody testing, which does not affect euploidy rate (121, 122); KIR–HLA-C genotyping, restricted to research use given inconsistent evidence (72, 123, 124); and serum vitamin D assessment, which may reasonably be included in a general health review but should not be offered as a specific RIF investigation, given the absence of consistent live-birth benefit from supplementation despite biological plausibility (125–127).

Non-evidence-based investigations increase costs, risk overdiagnosis, and may lead to misleading conclusions and unnecessary interventions. Such actions foster false hope, set unrealistic treatment expectations, and contribute to patient anxiety due to uncertain prognoses; the absence of supporting evidence can also result in inappropriate clinical actions that deviate from best practice (102, 128).

## Management: a five-tiered pragmatic algorithm

RIF does not arise from a single static causative factor but represents a dynamic clinical situation influenced by multiple, evolving factors. Physiological ageing, lifestyle changes, and the emergence of previously unrecognised pathologies across successive cycles necessitate an adaptive approach to management, guided by continual reassessment of contributory elements in each individual case. The five-tiered algorithm is presented in **Figure 3**.

**Figure 3:**
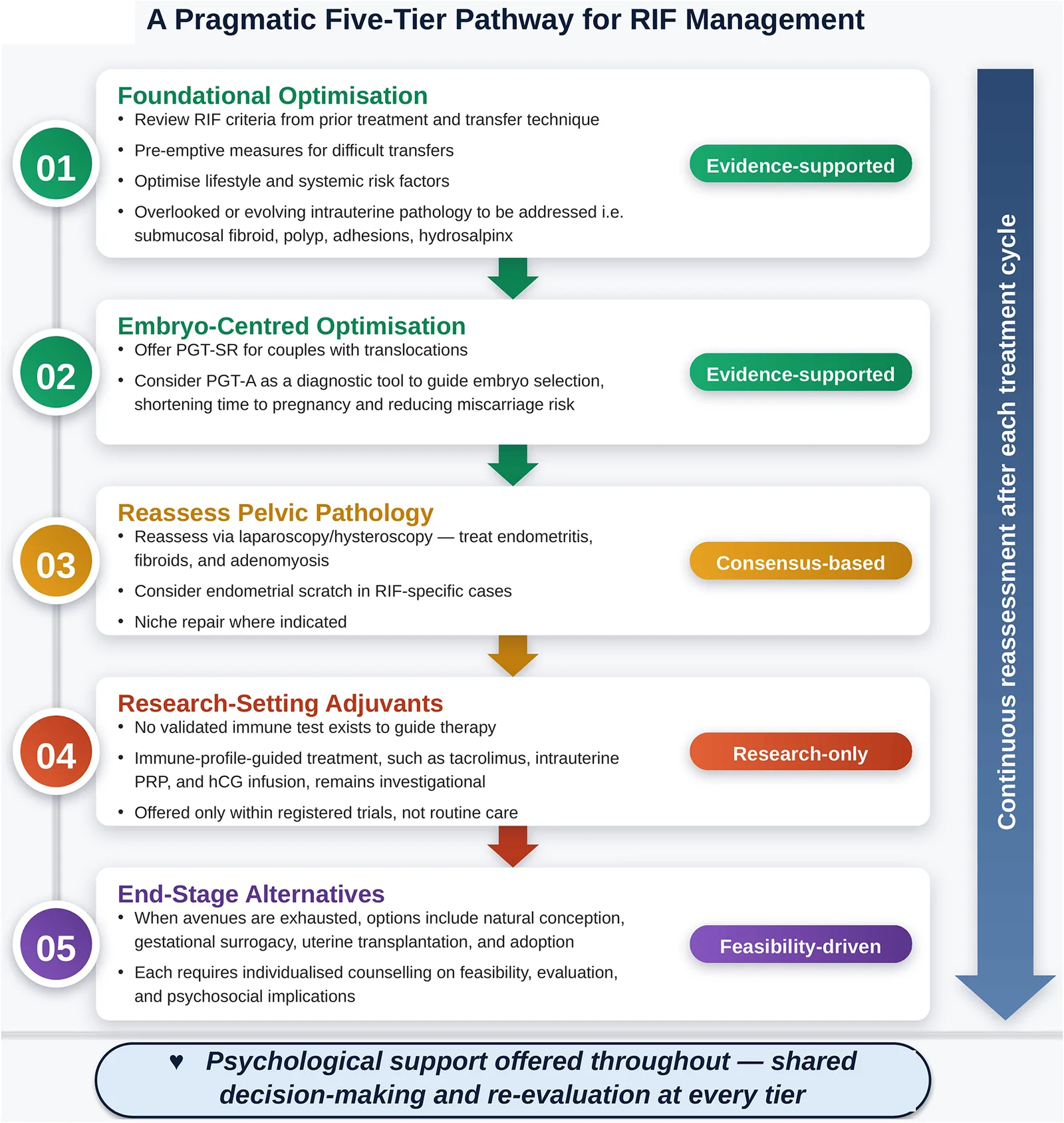
Five-tier, evidence-informed pathway for the evaluation and management of recurrent implantation failure (RIF), with continuous reassessment after each treatment cycle and psychological support offered throughout.

### First-tier management: foundational optimisation

This process begins with a thorough review of previous treatment cycles, confirming that embryos were of suitable morphological grade and the transfer technique was atraumatic and uncomplicated. A systematic review confirmed a dose-response relationship between embryo transfer difficulty and lower clinical pregnancy rates (129); pre-emptive measures for anticipated complex transfers include cervical priming, hysteroscopy, ultrasound guidance, and use of soft catheters, although harder catheters may be necessary for technically difficult transfers (130, 131). Transfer medium enriched with hyaluronic acid was associated with a higher pregnancy rate (52.2% versus 27.4%, P < 0.001) (132).

In parallel, modifiable lifestyle and systemic factors must be addressed (133, 134), including smoking cessation, stress reduction, weight optimisation, and treatment of active endometriosis or adenomyosis, with screening for thrombophilia reserved for patients with established risk factors (109, 135).

### Second-tier management: embryo-centred optimisation

For couples with chromosomal translocations, preimplantation genetic testing for structural rearrangements (PGT-SR) is indicated (94). By contrast, the role of PGT-A in RIF remains debated, although it is the only method capable of assessing embryonic chromosomal normality. Women experiencing RIF demonstrate higher aneuploidy rates (43–79%) than fertile controls, and true RIF occurs in fewer than 2% of patients after transfer of five euploid embryos (17).

PGT-A should be regarded primarily as a diagnostic rather than interventional tool (136); when previous cycles used untested embryos, PGT-A may assist in deselecting aneuploid embryos and directing management toward single versus double euploid transfer according to euploid rate, mosaicism, blastocyst expansion, and vitrification day, consistent with the reasoning of Cimadomo et al. and Kelly et al. (137, 138). Transferring euploid embryos can shorten time to pregnancy and reduce miscarriage risk (100).

### Third-tier management: re-evaluation of controversial pelvic pathologies

At this stage, conditions previously controversial or not fully addressed will be readdressed, with combined laparoscopy and hysteroscopy undertaken to identify and manage underlying pathologies such as endometriosis or chronic endometritis (90).

Chronic endometritis (CE) is present in approximately 30% of patients with RIF; screening via endometrial biopsy for plasma cell (CD138) positivity, followed by targeted antibiotic therapy, has been associated with higher implantation and live birth (139–142).

Non-cavity-distorting intramural fibroids of 2–6 cm significantly reduce live birth rates, though myomectomy has not shown a consistent live-birth benefit in comparative studies and may be reserved for large or multiple fibroids per British Fertility Society guidance (50, 143). Adenomyosis, particularly when involving the inner myometrium, is associated with reduced cumulative live-birth rates; therapeutic strategies include combined GnRH-agonist down-regulation with adjunctive letrozole prior to hormone replacement therapy (144, 145). In patients meeting ABCDE RIF criteria specifically — rather than in a general IVF population — an endometrial scratch performed approximately seven days before the frozen embryo transfer cycle may reasonably be discussed, followed by GnRH-agonist and hormone replacement therapy with intensive luteal support; the pivotal RCT in an unselected population was negative (146), but subgroup and meta-analytic evidence in women with previous IVF failure shows a significantly increased probability of live birth (RR 1.35, 95% CI 1.20–1.53; P < 0.001), with the greatest benefit in higher-order RIF (147). Caesarean scar niche repair may be considered where implantation remains unsuccessful and an isthmocele with intracavitary fluid is present, though long-term efficacy requires further investigation (148).

### Fourth-tier management: adjuvants in a research setting

A key limitation in reproductive immunology is the absence of validated immune testing and the inability to confirm whether immunotherapy restores endometrial function or corrects immune dysregulation, hindering assessment of true efficacy (149). Early uncontrolled data suggested benefit from tacrolimus in RIF; a subsequent single-centre, double-blind, placebo-controlled trial in immune-phenotyped women with elevated Th1/Th2 ratios reported improved clinical pregnancy (RR 2.83, 95% CI 1.30-6.44) and live-birth (RR 3.67, 95% CI 1.21–11.63) rates, though the trial was underpowered, did not incorporate PGT-A, and requires multicentre replication with euploid single embryo transfer as a mandatory design feature before adoption outside registered research settings (150, 151). Parallel work using immune-profile-guided treatment (the Utimpro test) reported higher pregnancy rates with treatment guided by profile, particularly among patients with ≥5 failed transfers, though this too requires further validation (124, 152). Intrauterine platelet-rich plasma and human chorionic gonadotrophin infusion remain confined to research settings pending robust evidence of efficacy (153).

### Fifth-tier management: end-stage alternatives

When all avenues are exhausted, alternatives include natural conception, gestational surrogacy, uterine transplantation, and adoption.

Natural conception after failed IVF is influenced by infertility cause and duration. Among women with RIF, the cumulative incidence of spontaneous livebirth over 5.5 years was 49%, with a median time to conception resulting in livebirth of nine months after diagnosis (154).

Gestational surrogacy offers a viable option for women with irreversible uterine factors, such as severe Asherman’s syndrome that may have been diagnosed after years of poor reproductive history, often following numerous unsuccessful hysteroscopic procedures or uncorrectable anomalies (155). Evidence confirms feasibility, safety, and psychosocial acceptability (156). Surrogacy requires exhaustive legal and medical evaluation, exclusion of reversible causes, multidisciplinary ethical review, and thorough counselling (157).

Uterine transplantation represents an evolving, limited-access option for women with uterine infertility, including RIF due to uterine factors; despite ethical and medical complexities, it provides a path to biological motherhood (158, 159).

Adoption may be considered once assisted reproductive options are exhausted, with counselling addressing process length, complexity, and psychosocial implications; outcomes are generally favourable, particularly when adoption occurs earlier in childhood (160, 161).

### Psychological support recommendation

All patients with RIF should be offered counselling support to process emotional impact and explore treatment continuation versus discontinuation decisions without judgment. Honest communication, empathy, and a holistic approach are just as important as algorithms and protocols. Shared decision-making, where we guide patients through uncertainty while respecting their values, is what defines us as physicians. In RIF, compassion is not an adjunct—it is the therapy.

## Discussion

### Strengths and contributions

This review demonstrates that the diagnosis of recurrent implantation failure is undermined at its foundation by a definitional disorder affecting more than 5,700 published studies, and that the ABCDE framework provides mathematically grounded, age-stratified, multi-registry-corroborated solution to this problem. Three contributions are interrelated: without the RIF/embryo-intrinsic-failure distinction, any threshold-based definition will be applied to a heterogeneous population that includes patients who have never actually tested their endometrium; without the serum βhCG endpoint, biochemical pregnancies are misclassified as successful implantation events; and without age-stratified thresholds anchored to an 80% CIR benchmark, the diagnosis of RIF remains statistically arbitrary.

The RIF/embryo-intrinsic-failure distinction merits further emphasis because its clinical consequences are immediate and measurable. Patients who have never transferred a qualifying blastocyst have not tested their implantation interface and should not receive an endometrial workup. This designation redirects investigation towards oocyte competence, laboratory standards, stimulation protocols, and sperm DNA integrity — the domains that genuinely determine whether a qualifying blastocyst can be produced. Clinicians who apply the ABCDE qualifying criteria before initiating a workup will, in practice, reclassify a substantial proportion of patients currently labelled as RIF, sparing them investigations with no mechanistic rationale in their clinical context. This reclassification is not merely academic: it prevents the psychological harm of repeated negative investigation, reduces unnecessary expenditure, and preserves the investigative pathway for patients who genuinely fulfil RIF criteria.

The principal strength of this review is the cross-registry corroboration of the ABCDE thresholds. The convergence of binomial calculations across SART, ANZARD, and ESHRE at identical thresholds for two of three age strata demonstrates the kind of cross-population biological consistency that gives a proposed framework genuine claim to broad applicability. The three clinical cases provide real-world illustration of the framework’s practical implications, including a compelling illustration of definitive RIF (Ms R.O.).

The 80% CIR anchor represents a considered consensus threshold supported by biological analogy with natural fertility (73) rather than a mathematically derived optimum; a ±5 percentage-point shift around the anchor itself (75% versus 85%) would shift the <35 and 35–37 thresholds by ±1 transfer — a worthwhile target for future independent validation. The framework closes a practical gap that existing proposals have not addressed (**Table 4**): bedside applicability without specialist statistical input, requiring only the patient’s age, the number of qualifying transfers, and confirmation of embryo quality and euploidy status. The compressed euploid criterion of two failed transfers is particularly important where PGT-A is performed selectively: once euploidy is confirmed, the principal source of statistical variation — embryo aneuploidy — has been removed, and a lower threshold is both mathematically and clinically defensible.

**Table 4:** Comparison of the ABCDE framework with existing RIF definitions.

Framework	CIR threshold/basis	Age-stratified?	Embryo quality requirement	Distinct euploid pathway?	Validation status
ESHRE Working Group (72)	60% CIR	No	“Viable” embryo (unspecified grade)	No	Consensus-based; not cross-registry corroborated
Lugano Workshop (18)	95% CIR	No	“Viable” embryo transfer	No	Consensus-based
Ata et al. (4)	Anticipated-aneuploidy-based	Yes (age-specific aneuploidy rates)	Blastocyst transfer	Implicit via aneuploidy modelling	Methodologically rigorous; not registry-corroborated
ABCDE (this review)	80% CIR (consensus-anchored)	Yes (4/5/6 by age band)	Good-quality blastocyst (≥3BB)	Yes — compressed to 2 transfers, all ages ≤40	Cross-registry corroborated; prospective validation pending

### Limitations

Four limitations merit explicit acknowledgement. First, the binomial model underpinning the ABCDE age-stratified thresholds assumes independent and identically distributed implantation probabilities across successive transfers; in practice, per-transfer implantation probability varies with embryo batch, endometrial preparation, and evolving clinical conditions, such that the derived thresholds represent pragmatic clinical heuristics rather than precise individual probability estimates. Second, cross-registry corroboration across SART, ANZARD, and ESHRE data demonstrates consistency of independently derived aggregate calculations; this is categorically distinct from, and does not substitute for, prospective validation in an independent individual-level cohort, which remains the essential next step before guideline adoption. Third, confidence intervals around the registry-derived per-transfer implantation rates are not available at the aggregate level, precluding formal individual-level uncertainty quantification pending prospective cohort data. Fourth, the registry datasets from which the thresholds were derived substantially under-represent East Asian, South Asian, and sub-Saharan African populations, and the three illustrative clinical cases derive from a single clinical centre; the generalisability of both the quantitative thresholds and the case-based illustrations to ethnically and clinically diverse populations therefore remains to be established. We further note that the ABCDE framework does not currently define a transfer-number threshold for women aged over 40, who are instead managed via individualised counselling regarding oocyte quality rather than a standardised RIF threshold. The literature search itself was limited to PubMed/MEDLINE and EMBASE, which may introduce an element of selection bias despite the breadth of the search strategy.

### Research priorities

Three near-term research priorities are ranked in order of urgency. First, and most urgent, prospective, multicentre, multi-ethnic international validation of the ABCDE thresholds — this is the most important single gap in the evidence base. A concrete design is proposed: a prospective, multicentre, multi-ethnic cohort study applying ABCDE criteria at the point of workup initiation, with cumulative live birth after reaching the relevant threshold as the primary endpoint, stratified by age band and registry/region, and powered to detect a clinically meaningful difference in cumulative live birth between ABCDE-positive patients and previously-labelled-RIF patients who do not meet ABCDE criteria; this design could additionally be extended to derive an age-band-specific threshold for women aged over 40 and to prospectively compare two- versus three-transfer euploid thresholds. Second, high-quality RCTs of tier-three interventions — specifically endometrial scratch, niche repair, and immune modulation — are required in ABCDE-defined RIF cohorts using euploid embryos and pre-specified live-birth primary endpoints. Third, non-invasive PGT-A using spent blastocyst culture media may reduce the embryonic burden of diagnosis within the near term. Looking further ahead, organoid-based endometrial modelling (162), artificial intelligence for patient stratification, and autologous stem cell applications will collectively enable the meta-analytic synthesis that has been impossible under the current definitional disorder.

To conclude, the findings of this review support the clinical translation of the ABCDE framework as a named, bedside-applicable, evidence-grounded definition of RIF, pending prospective validation. Prospective validation in diverse clinical cohorts and universal adoption of the framework as a standard case definition are now the critical next steps.
