# Supplementary material for: Recurrent implantation failure: a registry-corroborated, age-stratified definition and pragmatic five-tiered management algorithm
Source: Front Endocrinol (Lausanne). 2026 Oct 5;17:1930159. doi: 10.3389/fendo.2026.1930159 (PMC13678383; doi:10.3389/fendo.2026.1930159)
Supplement: Supplementary file 1 [file Table1.docx]

**Supplementary Table S1. Evidentiary basis underlying the diagnostic-utility assessments summarised in Table 3**

| **Investigation** | **Criterion** | **Table 3 assessment** | **Grade** | **Basis** | **Reference(s)** |
| --- | --- | --- | --- | --- | --- |
| **Uterine/vaginal microbiome** | Biological plausibility | **✗** | Not established | Not met — no reference cited for this investigation was identified as meeting this criterion (a coherent mechanistic pathway has been proposed in the literature) at the time of this review. | 103, 104 |
|  | Association | **✗** | Not established | Not met — no reference cited for this investigation was identified as meeting this criterion (at least one study reports a statistically significant relationship with implantation or pregnancy outcome) at the time of this review. | 103, 104 |
|  | Causation | **✗** | Not established | Not met — no reference cited for this investigation was identified as meeting this criterion (prospective or interventional evidence supports a causal rather than correlative relationship) at the time of this review. | 103, 104 |
|  | Threshold | **✗** | Not established | Not met — no reference cited for this investigation was identified as meeting this criterion (a clinically validated diagnostic cut-off exists) at the time of this review. | 103, 104 |
|  | Evidence | **✗** | Not established | Not met — no reference cited for this investigation was identified as meeting this criterion (supportive data derive from RCT or meta-analysis rather than observational study alone) at the time of this review. | 103, 104 |
|  | Reproducibility | **✗** | Not established | Not met — no reference cited for this investigation was identified as meeting this criterion (findings have been independently replicated) at the time of this review. | 103, 104 |
|  | Accuracy | **✗** | Not established | Not met — no reference cited for this investigation was identified as meeting this criterion (reported sensitivity/specificity or diagnostic performance meets an accepted standard) at the time of this review. | 103, 104 |
|  | Standardisation | **✗** | Not established | Not met — no reference cited for this investigation was identified as meeting this criterion (a validated, consensus laboratory or reporting protocol exists) at the time of this review. | 103, 104 |
| **NK cells** | Biological plausibility | **✓** | **Moderate** | Moderate. Refs 105 (Moffett & Shreeve, Hum Reprod 2015) and 106 (Lapides et al. 2023) both describe a proposed immunomodulatory role for uterine NK cells in placentation and trophoblast invasion, providing mechanistic rationale; both are narrative reviews rather than primary mechanistic data. | 105, 106 |
|  | Association | **✗** | Not established | Not met — no reference cited for this investigation was identified as meeting this criterion (at least one study reports a statistically significant relationship with implantation or pregnancy outcome) at the time of this review. | 105, 106 |
|  | Causation | **✗** | Not established | Not met — no reference cited for this investigation was identified as meeting this criterion (prospective or interventional evidence supports a causal rather than correlative relationship) at the time of this review. | 105, 106 |
|  | Threshold | **✗** | Not established | Not met — no reference cited for this investigation was identified as meeting this criterion (a clinically validated diagnostic cut-off exists) at the time of this review. | 105, 106 |
|  | Evidence | **✗** | Not established | Not met — no reference cited for this investigation was identified as meeting this criterion (supportive data derive from RCT or meta-analysis rather than observational study alone) at the time of this review. | 105, 106 |
|  | Reproducibility | **✗** | Not established | Not met — no reference cited for this investigation was identified as meeting this criterion (findings have been independently replicated) at the time of this review. | 105, 106 |
|  | Accuracy | **✗** | Not established | Not met — no reference cited for this investigation was identified as meeting this criterion (reported sensitivity/specificity or diagnostic performance meets an accepted standard) at the time of this review. | 105, 106 |
|  | Standardisation | **✗** | Not established | Not met — no reference cited for this investigation was identified as meeting this criterion (a validated, consensus laboratory or reporting protocol exists) at the time of this review. | 105, 106 |
| **ERA test** | Biological plausibility | **✗** | Not established | Not met — no reference cited for this investigation was identified as meeting this criterion (a coherent mechanistic pathway has been proposed in the literature) at the time of this review. | 107, 108 |
|  | Association | **✗** | Not established | Not met — no reference cited for this investigation was identified as meeting this criterion (at least one study reports a statistically significant relationship with implantation or pregnancy outcome) at the time of this review. | 107, 108 |
|  | Causation | **✗** | Not established | Not met — no reference cited for this investigation was identified as meeting this criterion (prospective or interventional evidence supports a causal rather than correlative relationship) at the time of this review. | 107, 108 |
|  | Threshold | **✗** | Not established | Not met — no reference cited for this investigation was identified as meeting this criterion (a clinically validated diagnostic cut-off exists) at the time of this review. | 107, 108 |
|  | Evidence | **✗** | Not established | Not met — no reference cited for this investigation was identified as meeting this criterion (supportive data derive from RCT or meta-analysis rather than observational study alone) at the time of this review. | 107, 108 |
|  | Reproducibility | **✗** | Not established | Not met — no reference cited for this investigation was identified as meeting this criterion (findings have been independently replicated) at the time of this review. | 107, 108 |
|  | Accuracy | **✗** | Not established | Not met — no reference cited for this investigation was identified as meeting this criterion (reported sensitivity/specificity or diagnostic performance meets an accepted standard) at the time of this review. | 107, 108 |
|  | Standardisation | **✗** | Not established | Not met — no reference cited for this investigation was identified as meeting this criterion (a validated, consensus laboratory or reporting protocol exists) at the time of this review. | 107, 108 |
| **Thrombophilia** | Biological plausibility | **✓** | **Moderate** | Moderate. General biological rationale for a thrombophilia–implantation link is discussed in the cited literature, though ref 109's own meta-analysis found no significant genetic association (see Association column). | 109, 110 |
|  | Association | **✗** | Not established | Not met — no reference cited for this investigation was identified as meeting this criterion (at least one study reports a statistically significant relationship with implantation or pregnancy outcome) at the time of this review. | 109, 110 |
|  | Causation | **✗** | Not established | Not met — no reference cited for this investigation was identified as meeting this criterion (prospective or interventional evidence supports a causal rather than correlative relationship) at the time of this review. | 109, 110 |
|  | Threshold | **✗** | Not established | Not met — no reference cited for this investigation was identified as meeting this criterion (a clinically validated diagnostic cut-off exists) at the time of this review. | 109, 110 |
|  | Evidence | **✗** | Not established | Not met — no reference cited for this investigation was identified as meeting this criterion (supportive data derive from RCT or meta-analysis rather than observational study alone) at the time of this review. | 109, 110 |
|  | Reproducibility | **✓** | **Moderate** | Moderate. Reflects the general reproducibility of standard clinical thrombophilia assays (e.g. Factor V Leiden, antiphospholipid panel) in laboratory medicine broadly; not directly demonstrated within refs 109–110 themselves. | 109, 110 |
|  | Accuracy | **✓** | **Moderate** | Moderate. As above — reflects general clinical-laboratory standards for thrombophilia assays rather than a diagnostic-accuracy figure reported in refs 109–110. | 109, 110 |
|  | Standardisation | **✓** | **Moderate** | Moderate. Thrombophilia panels are standardised in clinical laboratory medicine generally; this is broader medical-literature knowledge rather than something shown specifically in refs 109–110. | 109, 110 |
| **HLA-DQ** | Biological plausibility | **✓** | **Moderate** | Moderate. Maternal–fetal HLA recognition is a recognised mechanism in reproductive immunology, discussed in ref 111 (Varla-Leftherioti et al. 2010). | 111 |
|  | Association | **✗** | Not established | Not met — no reference cited for this investigation was identified as meeting this criterion (at least one study reports a statistically significant relationship with implantation or pregnancy outcome) at the time of this review. | 111 |
|  | Causation | **✗** | Not established | Not met — no reference cited for this investigation was identified as meeting this criterion (prospective or interventional evidence supports a causal rather than correlative relationship) at the time of this review. | 111 |
|  | Threshold | **✗** | Not established | Not met — no reference cited for this investigation was identified as meeting this criterion (a clinically validated diagnostic cut-off exists) at the time of this review. | 111 |
|  | Evidence | **✗** | Not established | Not met — no reference cited for this investigation was identified as meeting this criterion (supportive data derive from RCT or meta-analysis rather than observational study alone) at the time of this review. | 111 |
|  | Reproducibility | **✗** | Not established | Not met — no reference cited for this investigation was identified as meeting this criterion (findings have been independently replicated) at the time of this review. | 111 |
|  | Accuracy | **✗** | Not established | Not met — no reference cited for this investigation was identified as meeting this criterion (reported sensitivity/specificity or diagnostic performance meets an accepted standard) at the time of this review. | 111 |
|  | Standardisation | **✗** | Not established | Not met — no reference cited for this investigation was identified as meeting this criterion (a validated, consensus laboratory or reporting protocol exists) at the time of this review. | 111 |
| **Immune testing** | Biological plausibility | **✓** | **Moderate** | Moderate. Discussed within the ESHRE good practice recommendations on RIF (ref 72), a consensus document. | 72 |
|  | Association | **✗** | Not established | Not met — no reference cited for this investigation was identified as meeting this criterion (at least one study reports a statistically significant relationship with implantation or pregnancy outcome) at the time of this review. | 72 |
|  | Causation | **✗** | Not established | Not met — no reference cited for this investigation was identified as meeting this criterion (prospective or interventional evidence supports a causal rather than correlative relationship) at the time of this review. | 72 |
|  | Threshold | **✗** | Not established | Not met — no reference cited for this investigation was identified as meeting this criterion (a clinically validated diagnostic cut-off exists) at the time of this review. | 72 |
|  | Evidence | **✗** | Not established | Not met — no reference cited for this investigation was identified as meeting this criterion (supportive data derive from RCT or meta-analysis rather than observational study alone) at the time of this review. | 72 |
|  | Reproducibility | **✗** | Not established | Not met — no reference cited for this investigation was identified as meeting this criterion (findings have been independently replicated) at the time of this review. | 72 |
|  | Accuracy | **✗** | Not established | Not met — no reference cited for this investigation was identified as meeting this criterion (reported sensitivity/specificity or diagnostic performance meets an accepted standard) at the time of this review. | 72 |
|  | Standardisation | **✗** | Not established | Not met — no reference cited for this investigation was identified as meeting this criterion (a validated, consensus laboratory or reporting protocol exists) at the time of this review. | 72 |
| **Coeliac screening** | Biological plausibility | **✗** | Not established | Not met — no reference cited for this investigation was identified as meeting this criterion (a coherent mechanistic pathway has been proposed in the literature) at the time of this review. | 116–120 |
|  | Association | **✗** | Not established | Not met — no reference cited for this investigation was identified as meeting this criterion (at least one study reports a statistically significant relationship with implantation or pregnancy outcome) at the time of this review. | 116–120 |
|  | Causation | **✗** | Not established | Not met — no reference cited for this investigation was identified as meeting this criterion (prospective or interventional evidence supports a causal rather than correlative relationship) at the time of this review. | 116–120 |
|  | Threshold | **✗** | Not established | Not met — no reference cited for this investigation was identified as meeting this criterion (a clinically validated diagnostic cut-off exists) at the time of this review. | 116–120 |
|  | Evidence | **✗** | Not established | Not met — no reference cited for this investigation was identified as meeting this criterion (supportive data derive from RCT or meta-analysis rather than observational study alone) at the time of this review. | 116–120 |
|  | Reproducibility | **✗** | Not established | Not met — no reference cited for this investigation was identified as meeting this criterion (findings have been independently replicated) at the time of this review. | 116–120 |
|  | Accuracy | **✗** | Not established | Not met — no reference cited for this investigation was identified as meeting this criterion (reported sensitivity/specificity or diagnostic performance meets an accepted standard) at the time of this review. | 116–120 |
|  | Standardisation | **✓** | **Moderate** | Moderate. Coeliac serology (tTG-IgA) is a standardised, validated clinical assay in general medical practice; this reflects general laboratory-medicine knowledge rather than something shown specifically in the cited references. | 116–120 |
| **ANA & ATA** | Biological plausibility | **✓** | **Moderate** | Moderate. Autoimmune mechanisms in reproductive failure are plausibly described in refs 118 and 120 (thyroid autoimmunity reviews). | 116–122 |
|  | Association | **✗** | Not established | Not met — no reference cited for this investigation was identified as meeting this criterion (at least one study reports a statistically significant relationship with implantation or pregnancy outcome) at the time of this review. | 116–122 |
|  | Causation | **✗** | Not established | Not met — no reference cited for this investigation was identified as meeting this criterion (prospective or interventional evidence supports a causal rather than correlative relationship) at the time of this review. | 116–122 |
|  | Threshold | **✗** | Not established | Not met — no reference cited for this investigation was identified as meeting this criterion (a clinically validated diagnostic cut-off exists) at the time of this review. | 116–122 |
|  | Evidence | **✗** | Not established | Not met — no reference cited for this investigation was identified as meeting this criterion (supportive data derive from RCT or meta-analysis rather than observational study alone) at the time of this review. | 116–122 |
|  | Reproducibility | **✗** | Not established | Not met — no reference cited for this investigation was identified as meeting this criterion (findings have been independently replicated) at the time of this review. | 116–122 |
|  | Accuracy | **✗** | Not established | Not met — no reference cited for this investigation was identified as meeting this criterion (reported sensitivity/specificity or diagnostic performance meets an accepted standard) at the time of this review. | 116–122 |
|  | Standardisation | **✓** | **Moderate** | Moderate. ANA and thyroid antibody (TPOAb/TgAb) assays are standardised clinical laboratory tests generally; this reflects general laboratory-medicine knowledge rather than something demonstrated specifically in the cited references. | 116–122 |
| **Sperm DNA fragmentation** | Biological plausibility | **✓** | **Moderate** | Moderate. A plausible mechanistic role for sperm DNA integrity in embryo development is discussed in ref 112 (Llácer et al. 2025, SWOT analysis). | 112, 113, 114 |
|  | Association | **✗** | Not established | Not met — no reference cited for this investigation was identified as meeting this criterion (at least one study reports a statistically significant relationship with implantation or pregnancy outcome) at the time of this review. | 112, 113, 114 |
|  | Causation | **✗** | Not established | Not met — no reference cited for this investigation was identified as meeting this criterion (prospective or interventional evidence supports a causal rather than correlative relationship) at the time of this review. | 112, 113, 114 |
|  | Threshold | **✗** | Not established | Not met — no reference cited for this investigation was identified as meeting this criterion (a clinically validated diagnostic cut-off exists) at the time of this review. | 112, 113, 114 |
|  | Evidence | **✗** | Not established | Not met — no reference cited for this investigation was identified as meeting this criterion (supportive data derive from RCT or meta-analysis rather than observational study alone) at the time of this review. | 112, 113, 114 |
|  | Reproducibility | **✗** | Not established | Not met — no reference cited for this investigation was identified as meeting this criterion (findings have been independently replicated) at the time of this review. | 112, 113, 114 |
|  | Accuracy | **✗** | Not established | Not met — no reference cited for this investigation was identified as meeting this criterion (reported sensitivity/specificity or diagnostic performance meets an accepted standard) at the time of this review. | 112, 113, 114 |
|  | Standardisation | **✗** | Not established | Not met — no reference cited for this investigation was identified as meeting this criterion (a validated, consensus laboratory or reporting protocol exists) at the time of this review. | 112, 113, 114 |
| **Endometrial function test** | Biological plausibility | **✓** | **Moderate** | Moderate. Ref 115 (Lédée et al. 2023, narrative review) describes a mechanistic rationale for endometrial immune profiling and cites clinical studies over the past decade suggesting improved live-birth rates with personalised profiling, though the review itself does not report primary trial data with effect sizes. | 115 |
|  | Association | **✗** | Not established | Not met — no reference cited for this investigation was identified as meeting this criterion (at least one study reports a statistically significant relationship with implantation or pregnancy outcome) at the time of this review. | 115 |
|  | Causation | **✗** | Not established | Not met — no reference cited for this investigation was identified as meeting this criterion (prospective or interventional evidence supports a causal rather than correlative relationship) at the time of this review. | 115 |
|  | Threshold | **✗** | Not established | Not met — no reference cited for this investigation was identified as meeting this criterion (a clinically validated diagnostic cut-off exists) at the time of this review. | 115 |
|  | Evidence | **✗** | Not established | Not met — no reference cited for this investigation was identified as meeting this criterion (supportive data derive from RCT or meta-analysis rather than observational study alone) at the time of this review. | 115 |
|  | Reproducibility | **✗** | Not established | Not met — no reference cited for this investigation was identified as meeting this criterion (findings have been independently replicated) at the time of this review. | 115 |
|  | Accuracy | **✗** | Not established | Not met — no reference cited for this investigation was identified as meeting this criterion (reported sensitivity/specificity or diagnostic performance meets an accepted standard) at the time of this review. | 115 |
|  | Standardisation | **✗** | Not established | Not met — no reference cited for this investigation was identified as meeting this criterion (a validated, consensus laboratory or reporting protocol exists) at the time of this review. | 115 |
| **KIR-HLA genotype** | Biological plausibility | **✓** | **Moderate–Strong** | Moderate–Strong. The trophoblast HLA-C / maternal KIR interaction is a well-described mechanism in reproductive immunology (refs 123, 124). | 123, 124 |
|  | Association | **✓** | **Moderate** | Moderate. Ref 123 (Alecsandru et al. 2020, prospective cohort, n=204) found KIR AA genotype associated with significantly lower live birth rate and higher miscarriage risk with increasing fetal HLA-C2 load. Ref 124 (Seles et al. 2024, retrospective cohort, n=65) found immunomodulatory treatment significantly improved pregnancy rate in KIR AA patients (47.8% vs 23.7%, P=.008). Both are single-centre cohort studies; not yet independently replicated by a third group within this reference set. | 123, 124 |
|  | Causation | **✗** | Not established | Not met — no reference cited for this investigation was identified as meeting this criterion (prospective or interventional evidence supports a causal rather than correlative relationship) at the time of this review. | 123, 124 |
|  | Threshold | **✗** | Not established | Not met — no reference cited for this investigation was identified as meeting this criterion (a clinically validated diagnostic cut-off exists) at the time of this review. | 123, 124 |
|  | Evidence | **✗** | Not established | Not met — no reference cited for this investigation was identified as meeting this criterion (supportive data derive from RCT or meta-analysis rather than observational study alone) at the time of this review. | 123, 124 |
|  | Reproducibility | **✗** | Not established | Not met — no reference cited for this investigation was identified as meeting this criterion (findings have been independently replicated) at the time of this review. | 123, 124 |
|  | Accuracy | **✗** | Not established | Not met — no reference cited for this investigation was identified as meeting this criterion (reported sensitivity/specificity or diagnostic performance meets an accepted standard) at the time of this review. | 123, 124 |
|  | Standardisation | **✗** | Not established | Not met — no reference cited for this investigation was identified as meeting this criterion (a validated, consensus laboratory or reporting protocol exists) at the time of this review. | 123, 124 |
| **Vitamin D level** | Biological plausibility | **✓** | **Moderate** | Moderate. Refs 125 and 126 describe plausible immunomodulatory (NK cytotoxicity, Th1/Th2 balance) and endometrial-receptivity mechanisms for vitamin D in implantation. | 125–127 |
|  | Association | **✓** | **Moderate** | Moderate. Ref 125 (Chen et al. 2020, cohort, n=154) found significant differences in NK cytotoxicity and Th1 cytokine production by vitamin D status (P<.05). Ref 127 (Badihi et al. 2025, RCT, n=112) found combined vitamin D + vaginal probiotics significantly improved pregnancy rate vs control (46.4% vs 10.7%, P=.003) — the single strongest piece of evidence in this reference set, being an RCT with a live-outcome endpoint; note this positive result was for a combination intervention, not vitamin D alone. Ref 126 is an in vitro/animal mechanistic study, not clinical data. | 125–127 |
|  | Causation | **✗** | Not established | Not met — no reference cited for this investigation was identified as meeting this criterion (prospective or interventional evidence supports a causal rather than correlative relationship) at the time of this review. | 125–127 |
|  | Threshold | **✗** | Not established | Not met — no reference cited for this investigation was identified as meeting this criterion (a clinically validated diagnostic cut-off exists) at the time of this review. | 125–127 |
|  | Evidence | **✗** | Not established | Not met — no reference cited for this investigation was identified as meeting this criterion (supportive data derive from RCT or meta-analysis rather than observational study alone) at the time of this review. | 125–127 |
|  | Reproducibility | **✓** | **Limited–Moderate** | Limited–Moderate. Findings are directionally consistent across refs 125–127 but derive from different study designs and populations rather than independent replication of one defined finding. | 125–127 |
|  | Accuracy | **✗** | Not established | Not met — no reference cited for this investigation was identified as meeting this criterion (reported sensitivity/specificity or diagnostic performance meets an accepted standard) at the time of this review. | 125–127 |
|  | Standardisation | **✗** | Not established | Not met — no reference cited for this investigation was identified as meeting this criterion (a validated, consensus laboratory or reporting protocol exists) at the time of this review. | 125–127 |
